# Prolonged exposure to artificial light and carcinogenesis: A systematic review of oncostatic mechanisms associated with melatonin pathways

**DOI:** 10.1111/php.70040

**Published:** 2025-10-14

**Authors:** Gabriel Barboza, Jêmina Oliveira, Antônio Ferreira, Renan Lopes, Marli Cupertino

**Affiliations:** ^1^ Department of General Biology, Department of Medicine and Nursing Federal University of Viçosa (UFV) Viçosa Minas Gerais Brazil

**Keywords:** carcinogenesis, light at night, light pollution, melatonin

## Abstract

Light pollution from widespread artificial illumination affects photosensitive organisms, including humans. Prolonged exposure to artificial light at night (ALAN), particularly blue light, is associated with melatonin suppression and circadian disruption, both implicated in carcinogenesis. This systematic review investigated the relationship between extended ALAN exposure and malignant neoplasms, identifying associated cancer types and biological mechanisms. A search was conducted in PubMed/Medline and Scopus using PRISMA guidelines. Original studies evaluating associations between ALAN, light pollution, or blue light and cancer in humans were included. Eighteen studies demonstrated a positive link between ALAN and breast cancer, with mechanisms involving interference in the cell cycle, DNA repair, oxidative stress, and activation of oncogenic pathways. Night‐shift work correlated with increased breast cancer risk, reduced melatonin levels, and hormonal dysregulation. Exogenous melatonin showed oncostatic potential, reversing epigenetic changes induced by ALAN and reducing tumor burden. Melatonin suppression may promote tumor progression through circadian gene disruption and hormonal imbalance. While findings support a consistent link between ALAN exposure and oncogenesis—especially breast and prostate cancers—methodological variability and confounding factors, such as genetic predisposition and lifestyle, limit generalization. Further studies are needed to clarify mechanisms and explore preventive strategies, including light pollution control and melatonin‐based interventions.

## Graphical abstract

Exposure to artificial light at night (ALAN), particularly at blue wavelengths, disrupts circadian rhythms by suppressing melatonin, thereby increasing cancer susceptibility. This systematic review consolidates evidence linking prolonged ALAN exposure to an elevated risk of breast and prostate tumors. Underlying mechanisms include DNA damage, epigenetic dysregulation, oxidative stress, hormonal imbalance, and altered cell cycle control. Night‐shift work further exacerbates melatonin suppression and is associated with a higher cancer incidence. Conversely, melatonin exerts oncostatic effects, reversing ALAN‐induced molecular alterations and attenuating tumor progression.

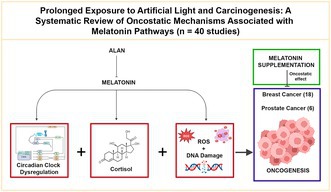

**Abbreviations**
AANAT: arylalkylamine N‐acetyltransferase
ACTH: adrenocorticotropic hormone
ALAN: artificial light at night
Apaf‐1: apoptotic protease‐activating factor 1
BAX: BAX protein
BMAL1: BMAL1 transcription factor gene
CAT: catalase
CLOCK‐BMAL: CLOCK‐BMAL1 complex
CRH: corticotropin‐releasing hormone
CYP1A1: cytochrome P450 family 1 subfamily A member 1
CYP1B1: cytochrome P450 family 1 subfamily B member 1
dATP: deoxyadenosine triphosphate
E‐BOX: enhancer box
FOXP3: forkhead box P3
GPx: glutathione peroxidase
GR: glucocorticoid receptor
IFN‐γ: interferon‐gamma
IL‐1: interleukin 1
IL‐12: interleukin 12
IL‐1β: interleukin 1 beta
IL‐2: interleukin 2
IL‐6: interleukin 6
IL‐8: interleukin 8
IpRGCs: intrinsically photosensitive retinal ganglion cells
LA: artificial light
LAN: nighttime artificial light
LANe: external artificial nighttime light
LANr: residential artificial nighttime light
LD: daylight
LNT: unspecified light exposure
MC2R: melanocortin receptor 2
MYC: proto‐oncogene Myc
NF‐κB: nuclear factor kappa B
NK: natural killer cells
NKG2D: NKG2D receptor
NS: not specified
p53: tumor suppressor protein p53
*Per1*
: Per1 gene
*Per2*
: Per2 gene
PGC‐1α: peroxisome proliferator‐activated receptor gamma coactivator 1‐alpha
PI3K/Akt/mTOR: PI3K/Akt/mTOR signaling pathway
PVN: paraventricular nucleus
REV‐ERB: orphan nuclear receptor REV‐ERB
RHT: retinohypothalamic tract
RORα: retinoic acid‐related orphan receptor alpha
ROS: reactive oxygen species
SCN: suprachiasmatic nucleus
SIRT1: silent information regulator 1
SOD: superoxide dismutase
TCD4: CD4 T cells
TCD8: CD8 T cells
TNF‐α: tumor necrosis factor‐alpha
Tregs: regulatory T cells
VEGF: vascular endothelial growth factor
γ‐H2AX: histone variant H2AX phosphorylated at serine 139

## INTRODUCION

The widespread incorporation of artificial light on a global scale has significantly transformed human exposure patterns to luminosity, leading to the emergence of light pollution, an environmental issue with social and biological consequences.^1^ This form of pollution, characterized by excessive or inappropriate artificial light emission, negatively impacts both the environment and photosensitive organisms, including humans. Nocturnal light exposure primarily occurs through electronic devices and artificial lighting, whether in indoor or outdoor environments.^2^

Recent studies suggest that excessive exposure to artificial light plays a relevant role in the development of malignant neoplasms. Life on Earth has evolved under the influence of a circadian cycle dictated exclusively by sunlight, characterized by high luminosity during the day and complete darkness at night. However, human activity has modified this natural pattern through the introduction of artificial lighting, resulting in daytime environments with relatively reduced luminous intensity compared to sunlight and nocturnal exposure to high‐intensity light sources. This alteration has become particularly significant with the widespread adoption of Light‐Emitting Diodes (LEDs), developed only 63 years ago, which emit a significant amount of radiation in the blue spectrum.^1^, ^3^

Continuous light exposure, especially at night, has been associated with changes in behavior, reproductive cycles, and hormonal regulation. In this context, one of the implicated mechanisms is the inhibition of the pineal gland and the consequent reduction in melatonin production, a hormone with oncostatic action, whose suppression may favor oncogenic processes.^4^ Moreover, disruptions in the circadian cycle, a crucial regulator of biological rhythms, are considered cancer‐promoting factors.^5^

Cancer is the second leading cause of death worldwide, accounting for approximately 9.6 million deaths in 2018, according to the World Health Organization (WHO). Breast cancer, the most prevalent among women, recorded 2.3 million new cases and 670,000 deaths in 2022, highlighting the importance of studying the relationship between artificial nighttime light exposure and breast oncogenesis, particularly concerning hormonal levels, estrogen, and melatonin.^6^ Increasing evidence indicates that exposure to artificial nighttime light (LAN) and the consequent disruption of the circadian rhythm, especially through melatonin suppression, may be associated with an increased risk of cancer. Furthermore, there are significant disparities in the burden and prognosis of the disease: in high Human Development Index (HDI) countries, 1 in 12 women will be diagnosed throughout their lifetime, and 1 in 71 will die from the disease; whereas in low‐HDI countries, although the number of diagnoses is lower, the relative mortality rate is higher, with 1 in 48 women dying from the disease.^6^, ^7^, ^8^ These data reinforce the importance of investigating environmental factors, such as LAN, in cancer etiology and the necessity of effective screening strategies and risk mitigation. This study aims to review scientific evidence from observational and experimental research in humans on the association between artificial light exposure and cancer development. Additionally, it seeks to identify the types of malignant neoplasms and explore the possible melatonin‐related biological mechanisms involved.

## MATERIALS AND METHODS

### Guiding question

What scientific evidence exists regarding the association between excessive artificial light exposure and the risk of developing malignant neoplasms in observational studies conducted in humans? Which types of malignant neoplasms are most frequently associated with specific light wavelengths? Finally, what biological mechanisms, specifically related to the oncostatic pathway of melatonin, have been proposed to explain the causal relationship between excessive artificial light exposure and carcinogenesis?

### Literature review

**FIGURE 1:**
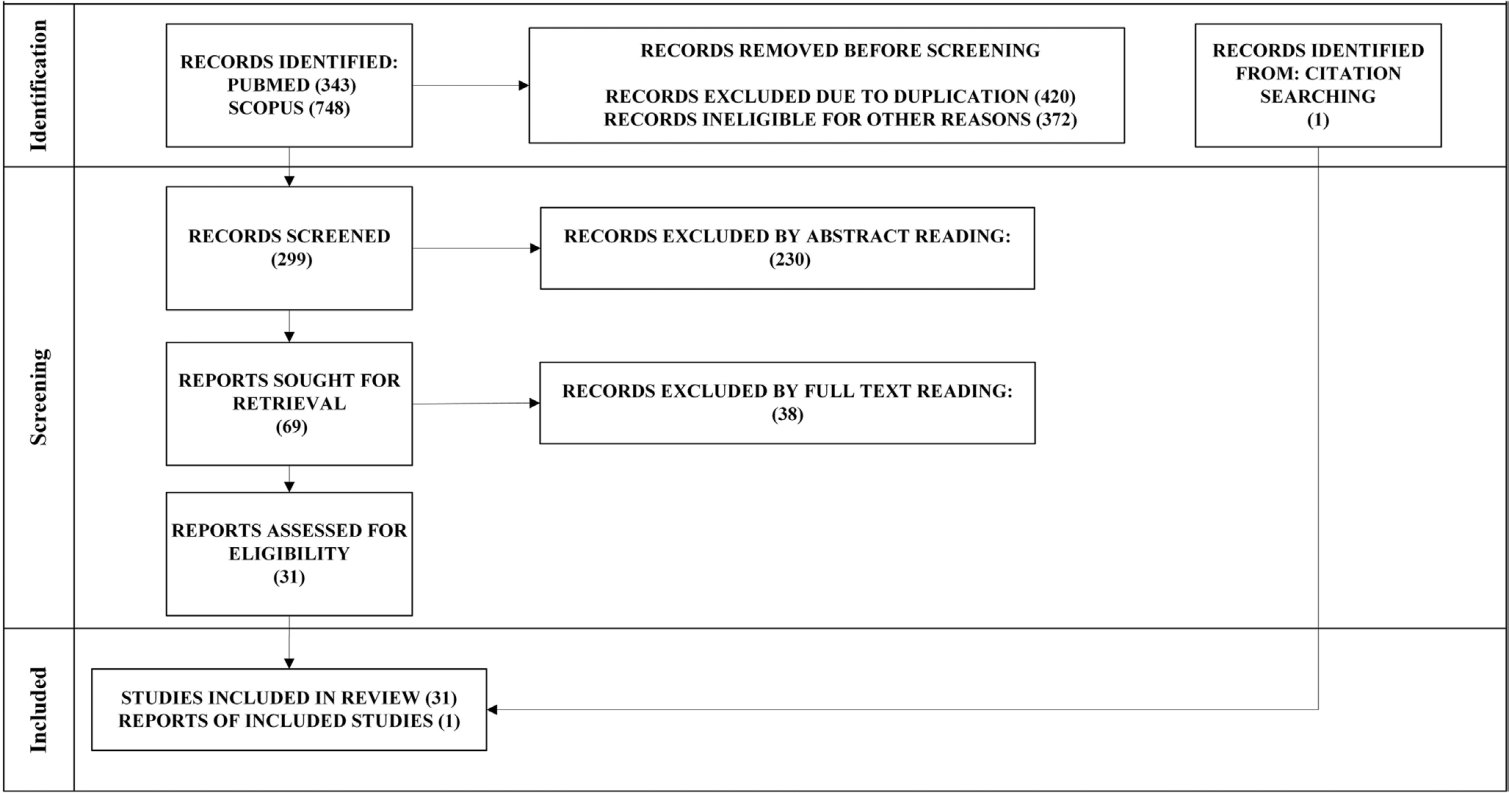
PRISMA flow diagram. The flowchart illustrates the research records obtained at each standardized stage of the search process necessary for conducting systematic reviews and meta‐analyses. Based on the PRISMA statement (http://www.prisma‐statement.org), accessed on November 23, 2024.

This study was conducted following the PRISMA guidelines (Figure 1), ensuring meticulous selection, extraction, and analysis of data. A comprehensive literature search was performed using the PubMed/Medline (https://pubmed.ncbi.nlm.nih.gov) and Scopus (https://www.scopus.com) databases. On November 21, 2024, an advanced search was conducted on these platforms. The search strategy encompassed two main approaches: direct searches in electronic databases and indirect screening of reference lists from the identified studies.

The search filters were based on the following combination of descriptors: ((Light at Night) OR (Blue Light) OR (Light Pollution)) AND ((Neoplasms) OR (Cancer)). In PubMed/Medline, the filters included standardized descriptors from the Medical Subject Headings (MeSH). These descriptors were adapted to meet the search requirements in Scopus (TITLE‐ABS‐KEY [descriptor]). No chronological restrictions were applied, and all original full‐text studies published up to 2024 were included in the review. The organization of terms and the results obtained are presented in Table 1.

**TABLE 1:** Search strategy used in the literature review and the results found in the databases.

Estratégia de busca (inglês)	PubMed	Scopus
(Light Pollution [Title/Abstract]) AND (Neoplasms [Title/Abstract])	0	39
(Light Pollution [Title/Abstract]) AND (Cancer [Title/Abstract])	41	89
(Light at night [Title/Abstract]) AND (Neoplasms [Title/Abstract])	3	193
(Light at night [Title/Abstract]) AND (Cancer [Title/Abstract])	270	352
(Light Pollution [Title/Abstract]) AND (Light at night [Title/Abstract]) AND (Cancer [Title/Abstract])	26	20
(Light Pollution [Title/Abstract]) AND (Light at night [Title/Abstract]) AND (Neoplasms [Title/Abstract])	0	47
(Light Pollution [Title/Abstract]) AND (Blue Light [Title/Abstract]) AND (Cancer [Title/Abstract])	3	8

Two reviewers conducted the initial search, removed duplicate articles, and selected titles and abstracts based on predefined eligibility criteria. The full texts of potentially relevant studies were then independently assessed by two reviewers. The degree of agreement between the reviewers during data selection and extraction was measured using the kappa test, yielding a value of 0.897. Any inconsistencies were resolved through consultation between the reviewers.

The Kappa index calculation was performed to assess the agreement between two researchers regarding the inclusion or exclusion of 85 fully reviewed articles. Of these, 30 articles were included in the final study, while 55 were excluded. The contingency table shows that both reviewers agreed on the inclusion of 30 articles and the exclusion of 51, while they disagreed on 4 cases. The observed agreement proportion (Po) was 0.953, representing the sum of agreements divided by the total number of evaluations. The expected agreement (Pe) was calculated based on marginal distributions, resulting in 0.543. Applying the Kappa index formula *κ* = (Po − Pe)/(1 − Pe), we obtained a value of 0.897.

### Study selection

To ensure the integrity and reliability of the review, a rigorous methodology for selection and analysis was implemented. Initially, two reviewers independently extracted data to eliminate potential biases. This independent analysis was essential to maintaining objectivity, particularly during the data collection and selection phases.

The inclusion criteria required that studies be original, observational, or experimental, published in full text, and investigate the association between artificial light exposure and the risk of cancer development in humans. The exclusion criteria were: (i) articles without full‐text availability; (ii) secondary studies (literature reviews, commentaries, letters to the editor, and editorials); (iii) studies that did not establish an association between excessive artificial light exposure and malignant neoplasms.

### Data extraction

Two independent reviewers extracted essential data, categorized into four descriptive levels: publication characteristics (author, year, and country), intervention details (control group, dose, frequency, route, and intervention duration), primary outcomes observed after treatment, and secondary outcomes. In case of disagreement regarding the extracted data, an additional reviewer participated in the discussion to resolve the issue.

### Complementary search on mechanistic pathways

In addition to the primary systematic search, a complementary review was performed to integrate recent experimental findings that elucidate the molecular mechanisms underlying melatonin's protective effects in the context of carcinogenesis. This additional search aimed to deepen the biological understanding of how melatonin modulates DNA repair, oxidative stress, apoptosis, and genomic stability, particularly under environmental stressors such as artificial light exposure. The search was conducted independently from the main dataset in the PubMed and Scopus databases using the following Boolean strategy: (Melatonin) AND (DNA Damage) AND (Cancer). Filters were applied to restrict the results to studies in animal models, and the search window was limited to publications from 2020 to 2025, ensuring the inclusion of recent and relevant data. Only original experimental articles were selected. The findings obtained through this complementary search were not subjected to the systematic selection process outlined in the PRISMA diagram, nor included in the kappa agreement calculation. Instead, they served to enrich the biological discussion presented in **“**Study limitations**”** section, contributing a mechanistic context to the observed associations between circadian disruption, melatonin suppression, and carcinogenesis.

## RESULTS

The initial search yielded 343 studies from PubMed/Medline and 748 from Scopus, totaling 1091 studies. After reviewing the titles and abstracts, 420 duplicates were removed, 372 studies were excluded for not being original articles, and another 230 studies were excluded for not establishing a relationship between artificial light exposure and malignant neoplasms, leaving 69 studies for full‐text review. Only 31 studies fully met the inclusion criteria, while 1 additional study was incorporated through indirect screening of reference lists, resulting in a total of 32 articles included in the systematic review (Figure 1).

The included studies were conducted in the United States (24.39%), Israel (13.56%), Russia, China, the United Kingdom, and South Korea (8.04% each), Spain (5.34%), the Netherlands, Japan, Germany, India, Australia, Slovakia, Poland, and Georgia (2.72% each). Additionally, a global study (Al‐Naggar and Anil, 2016) covering 158 countries accounted for 2.72% of the sample.

**FIGURE 2:**
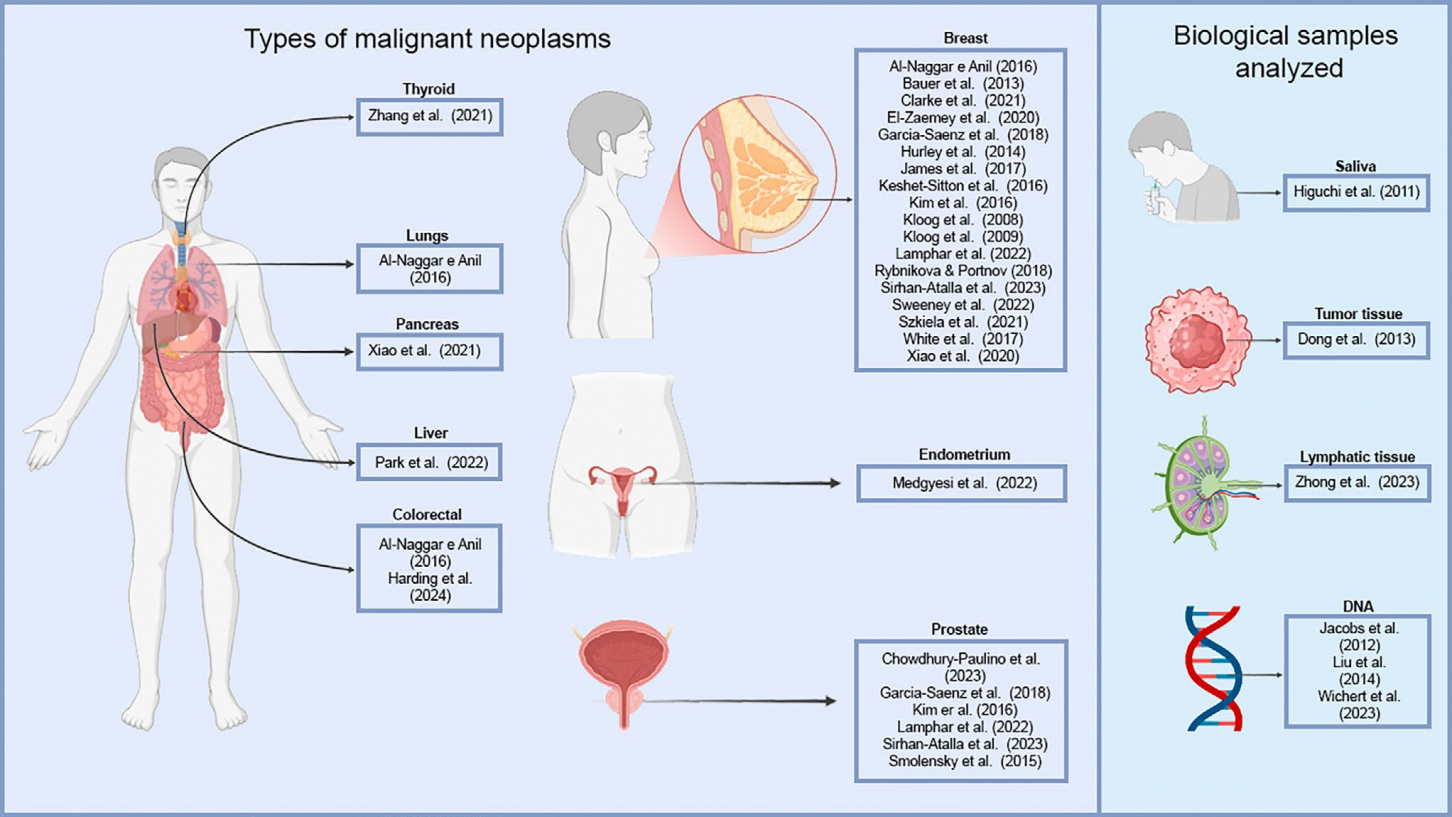
Types of malignant neoplasms associated with artificial light exposure (left). Biological tissues collected for biomarker analysis (right). The data in Table 2 compile information on the type of light, sample size, analyzed tissue, and possible experimental interventions extracted from the reviewed studies. In summary, only three studies did not demonstrate a positive relationship between excessive exposure to artificial light and carcinogenesis.

The reviewed studies analyzed human models exposed to artificial light at night (LAN). These studies investigated the relationship between nighttime light exposure and different types of cancer, as well as the collection of biological samples for biomarker analysis associated with the pathology (Figure 2). The most frequently studied cancers were breast cancer (59.38%), followed by prostate cancer (18.75%), colorectal cancer (6.25%), and liver, pancreas, lung, endometrial, and thyroid cancers (3.13% each). The primary biological samples used for biomarker analysis included neoplastic tissue biopsies, saliva, lymphoid tissue, and DNA.

The data in Table 2 compile information on the type of light, sample size, analyzed tissue, and possible experimental interventions extracted from the reviewed studies. In summary, only three studies did not demonstrate a positive relationship between excessive exposure to artificial light and carcinogenesis. Based on a large population‐based case–control study, it was concluded that night shift work is not associated with an increase in mammographic density.^9^ Similarly, Medgyesi et al. did not identify a significant relationship between nighttime artificial light exposure (LAN) and the risk of endometrial cancer. However, the authors emphasize the need for future investigations employing a more precise characterization of individual light exposure levels.^10^ Lastly, a study by Park et al. also found no association between outdoor residential LAN and hepatocellular carcinoma, suggesting that this lack of correlation may be partially attributed to methodological limitations in estimating light exposure.^11^

**TABLE 2:** Summary of studies on the effects of different types of light on humans.

Study	Type of light	Sample	*n*	Effect	Analyzed tissue	Intervention
Al‐Naggar e Anil (2016)	LAN	Populations from 158 countries	NS	Pro‐oncogenic	Breast, colorectal, lung	(−) Observational study
Bauer et al. (2013)	LNT	Women with breast cancer (Georgia)	48.511	Pro‐oncogenic	Breast	(−) Observational study
Chowdhury‐Paulino et al. (2023)	LANe	Men from the Health Professionals Study (USA)	51.529	Pro‐oncogenic	Prostate	(−) Observational study
Clarke et al. (2021)	LANe	16,941 Danish nurses	16.941	Pro‐oncogenic	Breast (Estrogen receptor‐positive)	(−) Observational study
Dong et al. (2013)	LAN	Human breast cancer cells	NS	Pro‐oncogenic	Breast cancer cells	(−) estudo in vitro
El‐Zaemey et al. (2020)	LANr	1.821 women	1.821	No association	Mammography	(−) Observational study
Garcia‐Saenz et al. (2018)	LANr + LANe	População da coorte MCC‐Spain (Espanha)	4.106	Pro‐oncogenic	Breast and prostate	(−) Observational study
Harding et al. (2024)	LANr	MCC‐Spain cohort population (Spain)	2.314	Pro‐oncogenic	Colorectal	(−) Observational study
Higuchi et al. (2011)	LNT	Young healthy men	11	Pro‐oncogenic	Saliva melatonin concentrations	(+) Controlled experimental study
Hurley et al. (2014)	LANr e LANe	106,731 women from the California Teachers Study cohort (USA)	106.731 (5.095 casos)	Pro‐oncogenic	Breast	(−) Observational study
Jacobs et al. (2012)	LAN	10 shift‐working women and 10 daytime workers	20	Pro‐oncogenic	DNA methylation	(−) Observational study
James et al. (2017)	LANe	Women from the Nurses' Health Study II (USA)	109.672	Pro‐oncogenic	Breast	(−) Observational study
Keshet‐Sitton et al. (2016)	LAN	110 breast cancer cases and 142 controls	252	Pro‐oncogenic (HIGHER URBAN RISK)	Breast	(−) Observational study
Kim et al. (2016)	LAN	General population (South Korea)	3.373.121	Pro‐oncogenic	Breast	(−) Observational study
Kim et al. (2016)	LAN	Population of Gwangju City and South Jeolla (South Korea)	NS	Pro‐oncogenic	Prostate	(−) Observational study
Kloog et al. (2008)	LANr	Women from the Israeli population	NS	Pro‐oncogenic	Breast	(−) Observational study
Kloog et al. (2009)	LAN	Population of Haifa (Israel)	NS	Pro‐oncogenic	Breast	(−) Observational study
Lamphar et al. (2022)	LAN	Human populations in Slovakia	NS	Pro‐oncogenic (BREAST) Anti‐oncogenic (PROSTATE)	Breast and prostate	(−) Observational study
Liu et al. (2014)	LAN	10 shift‐working women and 10 daytime workers	20	Pro‐oncogenic	DNA methylation	(−) Observational study
Medgyesi et al. (2022)	LAN	Adults (50–71 years, USA)	97.677	No association	Endometrial	(−) Observational study
Park et al. (2022)	LANe	Adults (50–71 years, USA)	451.945	No association	Hepatic	(−) Observational study
Rybnikova & Portnov (2018)	LA	Women with breast cancer (Israel)	NS	Pro‐oncogenic	Breast	(−) Observational study
Sirhan‐Atalla et al. (2023)	LAN + LD	Global data	NS	Pro‐oncogenic	Breast and prostate	(−) Observational study
Smolensky et al. (2015)	LAN + LD	Global data	NS	Pro‐oncogenic	Prostate and Melatonin concentrations	(−) Observational study
Sweeney et al. (2022)	LANr	Women from the Sister Study cohort (USA)	47.145	Pro‐oncogenic	Breast	(−) Observational study
Szkiela et al. (2021)	LANr	Night‐shift working women (Poland)	1.009	Pro‐oncogenic	Breast	(−) Observational study
White et al. (2017)	LANr	Women from the Sister Study Cohort (USA)	50.884	Pro‐oncogenic	Breast	(−) Observational study
Wichert et al. (2023)	GS	44,405 women (Breast Cancer Consortium)	44.405	Pro‐oncogenic	DNA polymorphisms	(−) Genetic analysis
Xiao et al. (2020)	LANr	Women from the NIH‐AARP Study (USA)	186.981	Pro‐oncogenic	Breast	(−) Observational study
Xiao et al. (2021)	LANr	Women from the NIH‐AARP Study (USA)	464.371	Pro‐oncogenic	Pancreatic	(−) Observational study
Zhang et al. (2021)	LANe	Adults (50–71 years, USA)	464.371	Pro‐oncogenic	Thyroid	(−) Observational study
Zhong et al. (2023)	LANe	Children (0–14 years, California)	141.882	Pro‐oncogenic	Lymphocytic leukemia	(−) Observational study

Abbreviations: GS, genetic study (SNPs in circadian genes); LA, artificial light; LAN, nighttime artificial light; LANe, external artificial nighttime light; LANr, residential artificial nighttime light; LD, daylight; LNT, unspecified light exposure; NS, not specified.

## DISCUSSION

This study analyzed research on the relationship between the oncostatic potential of melatonin, artificial light at night (ALAN), and carcinogenesis. In this context, melatonin, an essential component of the circadian system, plays fundamental roles in DNA repair, antioxidant defenses, inflammatory process control, and cell cycle regulation. However, nighttime light exposure suppresses its production, compromising these processes, which are crucial in preventing tumor initiation and progression. Polymorphisms in genes associated with melatonin synthesis, such as *TPH2* and *MAPK8*, were identified as having direct impacts on genomic stability and oncogenesis.^12^

Observational evidence reinforces this association. A study conducted in Slovakia found that cumulative exposure to light pollution over five years correlates positively with an increased incidence of breast cancer.^7^ Similarly, high nighttime light exposure has been linked to an increased risk of pancreatic ductal adenocarcinoma.^13^ These findings highlight the need for further investigation into the relationship between light pollution and the etiopathogenesis of different types of cancer.

Breast cancer is one of the tumors most frequently associated with circadian disruption. Studies indicate that ALAN affects critical molecular mechanisms of tumor control, particularly through interactions with circadian genes and hormones such as estrogen.^14^

Beyond its circadian production by the pineal gland, melatonin is also synthesized locally in various tissues, including the retina, gastrointestinal tract, skin, bone marrow, placenta, and immune cells. While pineal melatonin is rhythmically released into circulation and modulated by light through the inhibition of Arylalkylamine N‐acetyltransferase (AANAT), extrapineal melatonin primarily functions in an autocrine and paracrine manner, regulating processes such as cellular homeostasis, immunomodulation, and antioxidant defense. Circadian disruption induced by ALAN not only reduces systemic melatonin levels, thereby compromising its systemic oncostatic effects, but may also alter its local tissue production.

Regarding the molecular carcinogenic mechanisms of artificial light, the findings of the selected studies have been compiled into the following sections: (3.1) The Oncostatic Role of Melatonin in ALAN‐Associated Carcinogenesis, (3.2) ALAN‐Associated Breast Carcinogenesis: Molecular Mechanisms, and (3.3) Prostate Tumorigenesis and Other Cancers Associated with ALAN: Molecular Mechanisms—each of which will be presented in the subsequent sections.

### The oncostatic role of melatonin in ALAN‐associated carcinogenesis

The multiple functions of melatonin in pathways associated with gene regulation, its role as an antioxidant, and its involvement in inflammatory and immunogenic pathways establish it as a therapeutic agent, proving that it is much more than merely a sleep hormone synthesized by the pineal gland.^1^

In night‐shift workers, methylations in genes such as *DLX5*, *IGF2AS*, and *TP73* reinforce the epigenetic implications of LAN exposure.^15^ Similarly, increases in the methylation of *miR‐34b*, compromising antineoplastic immune processes, have also been demonstrated in night‐shift workers.^16^ Furthermore, melatonin administration may have a protective effect, reaffirming its oncostatic role.^17^ Additionally, associations between ALAN at shorter wavelengths and outdoor light exposure with carcinogenesis, particularly among premenopausal women.^18^, ^19^

However, some studies have found no association between night‐shift work and mammographic density, an important risk marker for breast cancer, suggesting that additional factors may influence this relationship.^9^ Moreover, it was highlighted that ALAN is associated with sleep disturbances and socioeconomic factors, which may also contribute to the increased risk of breast cancer.^20^, ^21^

**FIGURE 3:**
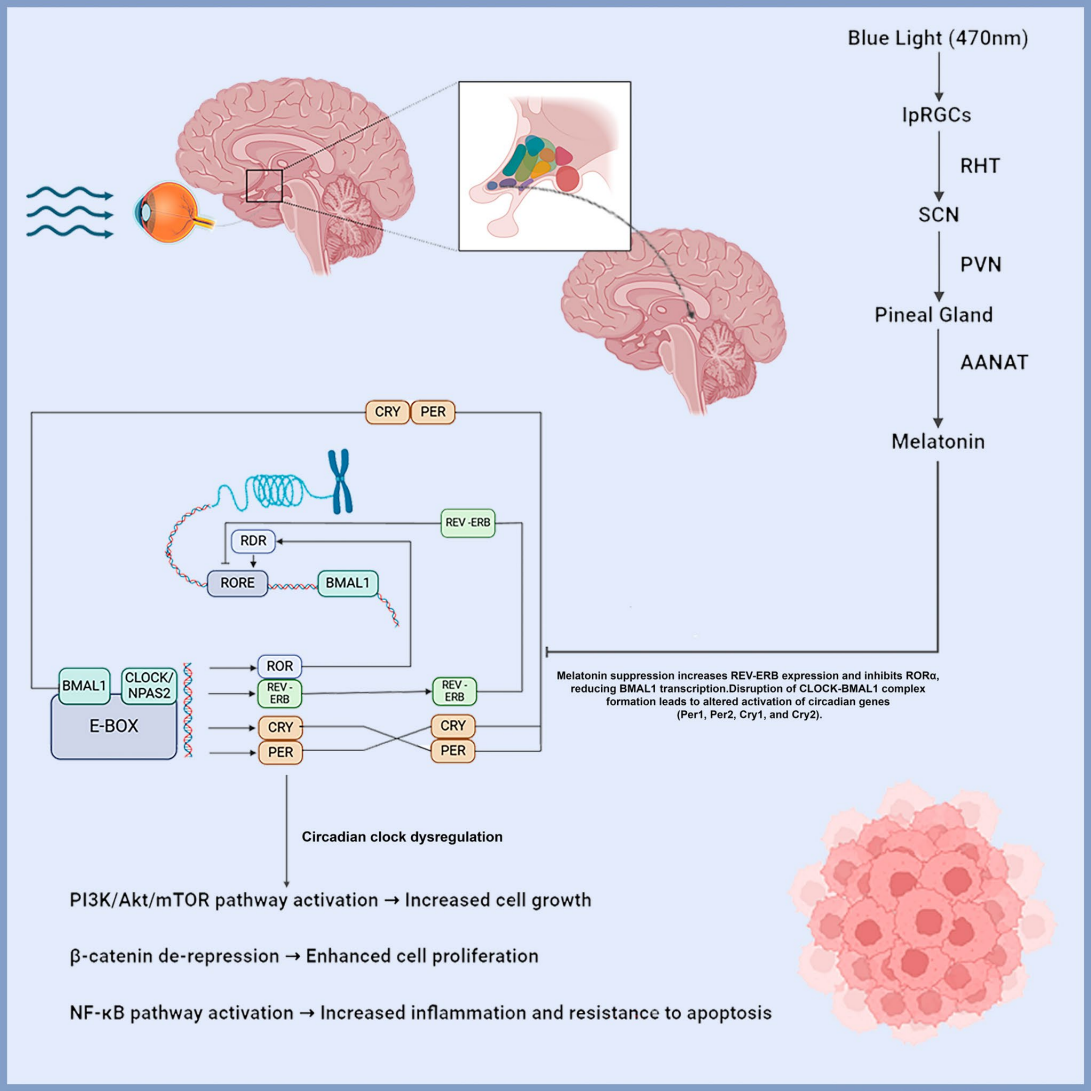
Mechanism of action of blue light associated with the oncostatic pathway of melatonin and genes linked to carcinogenesis. Blue light (470 nm) is one of the components of LAN, characterized by the shortest wavelength within the visible spectrum and the highest penetration capacity into organs and tissues. In this context, it exerts a particularly strong effect on melatonin suppression by activating intrinsically photosensitive retinal ganglion cells (ipRGCs). The light signal is transmitted through the retinohypothalamic tract (RHT) to the suprachiasmatic nucleus (SCN), which regulates the paraventricular nucleus (PVN) and inhibits pineal gland activity, thereby reducing melatonin synthesis via Arylalkylamine N‐acetyltransferase (AANAT). Chronic melatonin deficiency directly impacts the expression of circadian clock genes, such as BMAL1, Per1, Per2, Cry1, and Cry2, promoting circadian cycle dysregulation and fostering a pro‐tumorigenic environment. AANAT, arylalkylamine N‐acetyltransferase; BMAL1, BMAL1 transcription factor gene; CLOCK‐BMAL, CLOCK‐BMAL1 complex; *Cry1*, Cry1 gene; *Cry2*, Cry2 gene; E‐BOX, Enhancer Box; IpRGCs, intrinsically photosensitive retinal ganglion cells; NF‐κB, nuclear factor kappa B; *Per1*, Per1 gene; *Per2*, Per2 gene; PI3K/Akt/mTOR, PI3K/Akt/mTOR signaling pathway; PVN, paraventricular nucleus; REV‐ERB, Orphan nuclear receptor REV‐ERB; RHT, retinohypothalamic tract; RORα, retinoic acid‐related orphan receptor alpha; SCN, suprachiasmatic nucleus; β‐catenin, beta‐catenin.

Blue light (470 nm) is one of the components of LAN, characterized by the shortest wavelength within the visible spectrum and the highest penetration capacity into organs and tissues. In this context, it exerts a particularly strong effect on melatonin suppression by activating intrinsically photosensitive retinal ganglion cells (ipRGCs). The light signal is transmitted through the retinohypothalamic tract (RHT) to the suprachiasmatic nucleus (SCN), which regulates the paraventricular nucleus (PVN) and inhibits pineal gland activity, thereby reducing melatonin synthesis via Arylalkylamine N‐acetyltransferase (AANAT). Chronic melatonin deficiency directly impacts the expression of circadian clock genes, such as *BMAL1*, *Per1*, *Per2*, *Cry1*, and *Cry2*, promoting circadian cycle dysregulation and fostering a pro‐tumorigenic environment (Figure 3).

The mechanisms related to circadian cycle disruption and ALAN involve alterations in melatonin levels as well as genetic and epigenetic modifications. The genetic analysis of polymorphisms in genes associated with melatonin biosynthesis and signaling, as evidenced in the study by Wichert et al., revealed a significant association between genetic variations in genes such as *TPH2* and *MAPK8*, which directly influence the circadian rhythm.^12^ These polymorphisms may alter melatonin regulation and, consequently, affect cellular responses to stress and DNA repair. Inadequate regulation of these processes is closely linked to genomic instability, one of the primary facilitators of carcinogenesis.^12^

Genomic instability results in mutations that can activate oncogenes or deactivate tumor suppressor genes, fostering an environment conducive to the development of neoplastic cells. Circadian dysregulation induced by LAN compromises melatonin secretion and disrupts biological rhythms, directly impacting immune surveillance and cellular repair mechanisms. The interference with melatonin contributes to a state of chronic microinflammation, promoting tumor progression. Melatonin not only regulates circadian rhythms but also modulates the immune system, serving as a crucial regulator of inflammatory responses. Its chronic suppression impairs the body's ability to detect and eliminate mutant cells, facilitating tumor development.^12^

Higuchi et al. also provide relevant data on how melatonin modulation may influence carcinogenesis, particularly through its impact on sleep and immune vigilance. Additionally, blue light, which has an especially intense effect on melatonin suppression, has been associated with alterations in the function of central nervous system cells, with potential repercussions on the regulation of biological processes essential for cellular homeostasis.^22^

Studies have highlighted melatonin's ability to combat oxidative stress, either as a free radical scavenger or through the activation of antioxidant enzymes. Melatonin is involved in pathways that maintain the oxidative/antioxidant balance of the body, offering protective effects against induced oxidative stress—for instance, oxidative damage caused by non‐ionizing electromagnetic fields—through its radical‐scavenging activity, by reducing the singlet‐triplet conversion rate of radical pairs, and by decreasing the concentration of triplet products.^23^

**FIGURE 4:**
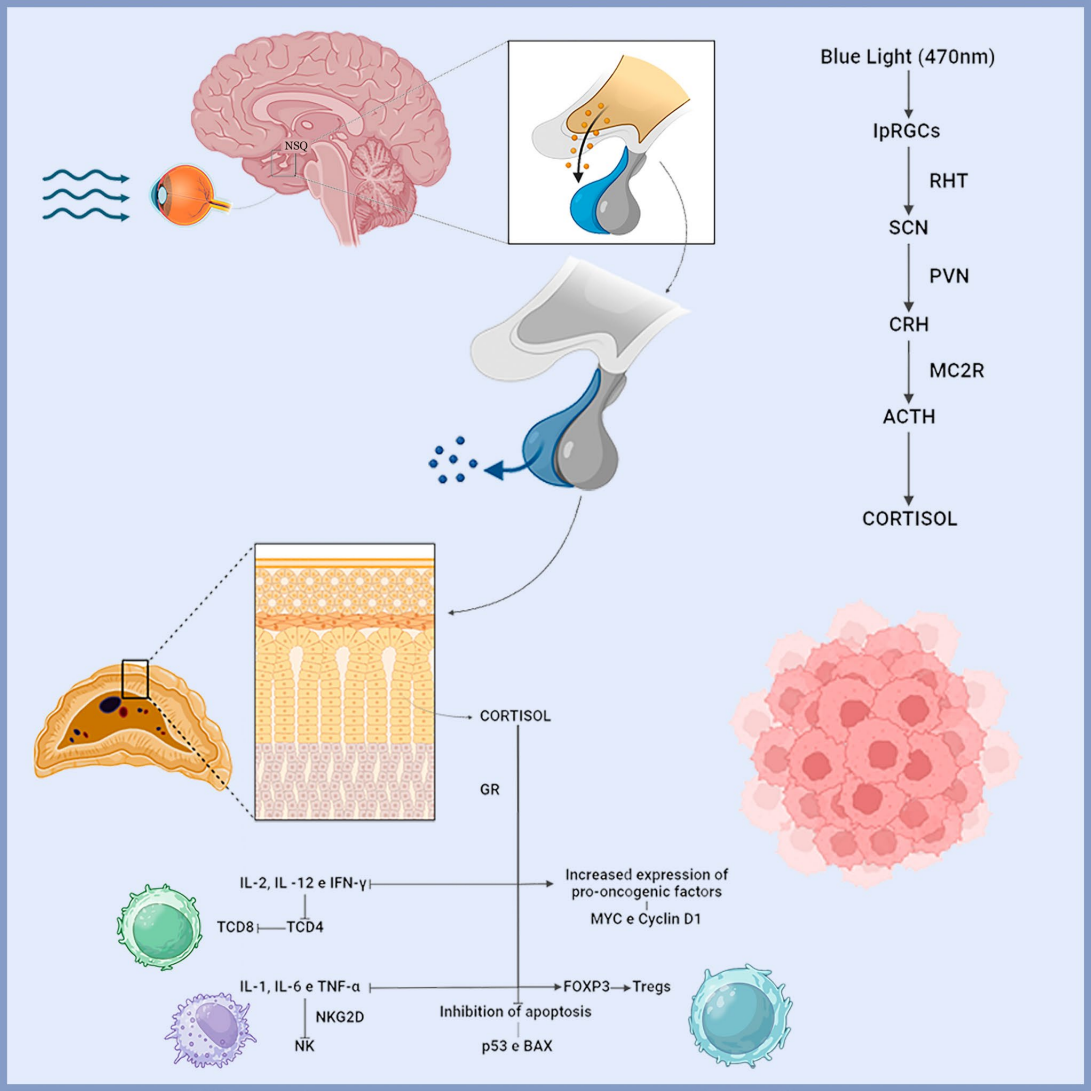
Activation of the hypothalamic–pituitary–adrenal (HPA) axis by blue light and its immunosuppressive and pro‐oncogenic effects. This figure, exposure to blue light (470 nm) activates the hypothalamic–pituitary–adrenal (HPA) axis, promoting cortisol release. This hormone negatively impacts immune response by modulating the expression of inflammatory cytokines and reducing the activity of CD8+ T cells, CD4+ T cells, and natural killer (NK) cells. Additionally, cortisol contributes to a pro‐oncogenic environment by increasing the expression of genes such as *MYC* and *Cyclin D1* while inhibiting apoptosis through the regulation of *p53* and *BAX*. These mechanisms, combined with melatonin suppression, facilitate tumor progression. ACTH, adrenocorticotropic hormone; BAX, BAX protein; CRH, corticotropin‐releasing hormone; FOXP3, forkhead box P3; GR, glucocorticoid receptor; IFN‐γ, interferon‐gamma; IL‐1, interleukin 1; IL‐12, interleukin 12; IL‐2, interleukin 2; IL‐6, interleukin 6; IpRGCs, intrinsically photosensitive retinal ganglion cells; MC2R, melanocortin receptor 2; MYC, proto‐oncogene Myc; NK, natural killer cells; NKG2D, NKG2D receptor; p53, tumor suppressor protein p53; PVN, paraventricular nucleus; RHT, retinohypothalamic tract; SCN, suprachiasmatic nucleus; TCD4, CD4 T cells; TCD8, CD8 T cells; TNF‐α, tumor necrosis factor‐alpha; Tregs, regulatory T cells.

As illustrated in Figure 4, exposure to blue light (470 nm) activates the hypothalamic–pituitary–adrenal (HPA) axis, promoting cortisol release. This hormone negatively impacts immune response by modulating the expression of inflammatory cytokines and reducing the activity of CD8+ T cells, CD4+ T cells, and natural killer (NK) cells. Additionally, cortisol contributes to a pro‐oncogenic environment by increasing the expression of genes such as *MYC* and *Cyclin D1* while inhibiting apoptosis through the regulation of *p53* and *BAX*. These mechanisms, combined with melatonin suppression, facilitate tumor progression.

Furthermore, it highlighted the impact of LAN on the methylation of genes such as *DLX5* and *TP73*, both associated with an increased risk of tumor development.^15^ Genomic instability leads to mutations that can activate oncogenes or deactivate tumor suppressor genes, fostering an environment conducive to the development of neoplastic cells. Epigenetic modifications, such as those identified by Jacobs et al., also demonstrated that prolonged LAN exposure and shift work are associated with significant changes in DNA methylation, affecting genes like *DLX5*, *IGF2AS*, and *TP73*, thereby potentially amplifying the risks of carcinogenesis.^15^

Beyond genetic factors, we observed an association between LAN and a significant increase in cancer risk, particularly when considered alongside other environmental factors such as air pollution and population density.^24^

### ALAN‐associated breast carcinogenesis: Molecular mechanisms

Growing evidence suggests that circadian cycle dysregulation, particularly in individuals engaged in night shift work, may significantly increase the risk of developing breast neoplasms.^15^, ^16^, ^25^ Prolonged exposure to shift work significantly increased the methylation of the *miR‐34b* promoter, potentially reducing its antineoplastic function in female shift workers compared to daytime workers. This process may weaken the immune‐mediated ability of the body to combat carcinogenesis.^15^

These findings reinforce those reported by Jacobs et al., who compared long‐term night shift workers with daytime workers regarding gene methylation in DNA samples. They observed higher methylation in 20 genes among long‐term shift workers and reduced methylation in 30 genes in women who only worked during the day.^15^ The genes *DLX5*, *IGF2AS*, and *TP73* exhibited methylation in multiple CpG sites and were the most affected by shift work. These alterations suggest epigenetic mechanisms through which night shift work could be associated with breast cancer.^15^

Dong et al. reported significant epigenetic changes, highlighting the expression of mRNA for genes involved in phototransduction—*PDE6B*, *PDE6C*, and *PDE6D*—in breast tumor cells.^25^ Additionally, the study observed the relevant expression of circadian genes, particularly the presence of *PDE6B* in MCF‐7 tumor cells. The findings suggest that the regulation of circadian genes, such as *PERIOD 2*, *CLOCK*, *TIMELESS*, *CRYPTOCHROME 1*, and *CRYPTOCHROME 2*, may be associated with the expression control of *PDE6* genes, contributing to the pathophysiology of light influence on breast cancer development.^25^

In line with these findings, we observed a statistically significant association between breast cancer and ALAN (*p* < 0.01), with a stronger correlation to short‐wavelength blue/green subspectrum light.^19^ Clarke et al. classified melatonin as an oncostatic hormone.^17^ The relevant melatoninergic properties prevent breast carcinogenesis, citing their antioxidant, apoptotic, and antiproliferative effects on cancer cells.^26^ Furthermore, they delineated the influence of LAN from both internal and external sources, both of which were associated with breast malignancy. The authors also observed a small but significant association between sleeping with a light source present in the bedroom or with televisions left on and breast cancer.^26^

White et al. reaffirmed the effects of indoor LAN by evaluating sleep characteristics that could be linked to nighttime light exposure and breast carcinogenesis, such as the type of light to which participants were exposed during sleep, the act of turning on a light at night, and nighttime awakenings.^27^ Sleep characteristics themselves were not associated with breast cancer risk, including sleep duration. However, quality‐related factors—such as difficulty sleeping, the presence of light or a television in the bedroom, and reduced sleep duration relative to individual needs—were linked to the malignant tumor in question, particularly in estrogen receptor‐positive (ER+) cases.^27^ These findings are supported by Xiao et al., who further explored the relationship between sleep disturbances and increased LAN exposure, demonstrating its role in intensifying the influence of nighttime light exposure on breast carcinogenesis.^13^

A 10% higher risk of breast cancer was observed, with an even stronger association in ER+ cases among postmenopausal women in the highest quintile of ALAN exposure.^21^ Areas with the highest ALAN exposure were predominantly metropolitan regions with high population densities. A correlation was observed between inadequate sleep patterns and higher levels of external light exposure, a condition often linked to unfavorable socioeconomic contexts. This finding underscores the social risks that, alone or in combination with individual habits, contribute to greater LAN exposure and the development of breast cancer, consistent with studies that have demonstrated a significant association between ALAN, alcohol consumption, and breast cancer.^20^

A positive relationship between breast cancer and outdoor nighttime light exposure based on an adjusted statistical model. Using this model, the authors calculated the relative risk, expressed as the Hazard Ratio (HR), for developing the tumor in relation to increasing nighttime light incidence.^18^ For each interquartile range increase of 31.6 nW/cm^2^/sr in average outdoor LAN exposure, the estimated HR was 1.05. According to the authors, residential artificial outdoor light exposure at night may increase the risk of invasive breast cancer, particularly among premenopausal women and smokers.^18^ These findings align with those of Hurley et al., who reported an increased risk of breast cancer among women living in areas with higher nighttime outdoor light exposure, especially among premenopausal individuals.^28^

Furthermore, night shift work was identified as a significant risk factor for breast cancer, ranking third after high body mass index (BMI) and nonexistent or short‐duration breastfeeding.^29^ Night shift work increases malignancy risk by more than twofold (OR = 2.2; 95% CI 1.57–3.08) and escalates with greater intensity of night shift work, supporting the hypothesis of its harmful effects, which may contribute to an increased risk of developing breast cancer.^29^

Conversely, no association was found between mammographic density—defined as a radiographic opacity of epithelial and stromal tissue—and night shift work.^9^ Mammographic density is considered one of the strongest risk factors for breast cancer and a key marker for prevention and intervention. Although the authors acknowledged the pathways through which shift work could be linked to breast malignancy, including LAN exposure, sleep disorders, and sleep disruption, they found no association between shift work‐related factors and increased mammographic density in the evaluated group.^9^

The Study investigated the relationship between breast cancer and LAN at a population level. The study revealed a 73% higher incidence of breast neoplasia in Israeli communities with the highest levels of nighttime brightness compared to those with the lowest levels. A strong positive association between LAN and breast cancer was observed (*p* < 0.05). The authors suggest that these findings should be analyzed in light of the impact of artificial lighting on the suppression of melatonin production and secretion by the pineal gland, highlighting its influence on hormonal pathways related to the development of malignant breast tumors.^30^

A higher prevalence of breast cancer in women exposed to ALAN, in both urban and rural areas of South Korea. An ecological analysis was conducted in specific regions, calculating the prevalence per locality and its association with LAN levels. The results revealed an 11.4% higher prevalence of breast cancer in areas with greater nighttime brightness, even after adjustments for risk factors such as obesity and smoking.^20^

In a related finding, an investigation of breast cancer incidence in different settings found that in urban areas, nighttime cellphone use and proximity to artificial light sources increased tumor risk. High levels of exposure to artificial light, both indoors and outdoors, correlated with a higher prevalence of cancer, regardless of whether the environment was rural or urban. Conversely, longer sleep duration and reading before bedtime were associated with a lower risk.^31^

### Prostate tumorigenesis and other cancers associated with ALAN: Molecular mechanisms

Beyond breast cancer, ALAN has also been associated with prostate cancer and other tumor types. In a meticulously conducted study in South Korea, it was demonstrated that light at night (LAN), when analyzed within an urban context, significantly increases the incidence of prostate cancer, even after strict adjustments for confounding factors such as smoking and alcohol consumption.^32^ This analysis reinforces the hypothesis that urbanization—mediated by circadian disruption induced by LAN—plays a central role in prostate oncogenesis. Complementarily, other researchers investigated the effects of LAN on biological rhythms, highlighting the reduction in melatonin production and vitamin D deficiency as key contributors to prostate tumor progression.^33^ Daylight deprivation, in synergy with LAN, alters not only circadian biomarkers but also modulates the tumor microenvironment in a manner conducive to carcinogenesis.

A robust case–control study conducted across 11 regions of Spain demonstrated that exposure to the blue spectrum of LAN significantly increases the risk of prostate cancer (OR = 2.05), representing an alarming environmental threat.^34^ On a more global scale, LAN has been unequivocally associated with increased age‐adjusted incidence rates of various cancers—including lung, colorectal, and prostate—indicating that this environmental factor transcends geographical boundaries and socioeconomic differences, acting as a universal carcinogenic agent.^35^ Although a recent prospective analysis involving over 49,000 participants did not find a significant cumulative association between LAN and overall or fatal prostate cancer risk, their findings revealed critical nuances: a positive correlation between initial LAN exposure and prostate cancer incidence in geographically stable individuals suggests that the effects of LAN may be mediated by specific behavioral and genetic factors, which warrant further investigation.^36^

Other malignancies also demonstrate susceptibility to LAN. A strong association has been identified between LAN and thyroid cancer—particularly the papillary subtype—most notably at early stages and among women.^37^ This finding suggests a potential selective effect of LAN on hormone‐sensitive tumors, underscoring the importance of investigating the underlying molecular pathways. In contrast, while examining endometrial cancer, we did not observe direct associations but proposed that the interplay of hormonal and metabolic factors may attenuate or obscure the effects of LAN in this context.^10^ Studies on colorectal cancer highlighted the superiority of advanced satellite‐based technologies in estimating LAN exposure, pointing to methodological refinement as a critical necessity for future studies.^38^ No association was found between external LAN and liver cancer or hepatocellular carcinoma, suggesting that limitations in LAN exposure assessment may have influenced their findings and underscoring the importance of incorporating exposure duration in future research.^11^

Further expanding the investigative scope, LAN exposure has been correlated with acute lymphoblastic leukemia in Hispanic children, emphasizing the relevance of interactive genetic and environmental factors.^39^ These findings, alongside research linking LAN to hormone‐related cancers and obesity, reinforce the central role of melatonin as a key pathophysiological mediator. Its dysfunction may trigger molecular cascades involving uncontrolled cellular proliferation and apoptotic resistance.^40^

### Circadian disruption, DNA damage, and genomic instability: Underlying mechanisms of ALAN‐associated carcinogenesis

Although previous sections have explored the theoretical and epidemiological basis for the association between ALAN exposure and carcinogenesis, it is important to emphasize that no studies have directly investigated the relationship between ALAN and genomic instability or DNA damage. Nevertheless, studies addressing the role of melatonin in this context have been identified. Given that ALAN suppresses melatonin production, the mechanisms linking melatonin to DNA damage and genomic instability are examined herein.

Genomic instability, characterized by spontaneous mutations, chromosomal aberrations, and impaired DNA repair mechanisms, constitutes a fundamental driver of tumor initiation and progression. Evidence indicates that exposure to artificial light at night (ALAN) induces circadian disruption, melatonin suppression, and compromised antioxidant defenses, collectively fostering a cellular environment conducive to mutagenesis and neoplastic transformation.^41^

Mitochondrial dysfunction represents a pivotal upstream event in the initiation of the intrinsic apoptotic signaling cascade. This pathway is primarily mediated by the permeabilization of the mitochondrial outer membrane, a process facilitated by the accumulation of reactive oxygen species (ROS) and the modulation of Bcl‐2 family proteins. Permeabilization of the mitochondrial outer membrane enables the release of apoptogenic factors into the cytosol, most notably cytochrome c. In the cytoplasm, cytochrome c associates with apoptotic protease activating factor‐1 (Apaf‐1) and dATP to form the apoptosome complex, which subsequently facilitates the autocatalytic activation of caspase‐9. Activated caspase‐9 cleaves and activates downstream executioner caspases, particularly caspase‐3 and caspase‐7, culminating in the proteolytic degradation of key cellular substrates, DNA fragmentation, and morphological disassembly consistent with programmed cell death. This tightly regulated mechanism is essential for maintaining genomic stability and eliminating cells harboring irreparable damage or neoplastic potential.^42^, ^43^

Concurrently, the nuclear factor kappa‐light‐chain‐enhancer of activated B cells (NF‐κB) signaling pathway functions as a central transcriptional regulator of cellular homeostasis in response to oxidative, inflammatory, and genotoxic insults. In its inactive state, NF‐κB dimers are retained in the cytoplasm through interaction with inhibitory IκB proteins. Activation of the pathway involves phosphorylation of IκB by the IκB kinase (IKK) complex, leading to its ubiquitination and subsequent proteasomal degradation. The liberated NF‐κB dimers translocate into the nucleus, where they bind to κB consensus sequences in the promoter regions of target genes. This binding induces transcription of genes associated with inflammatory mediators, anti‐apoptotic proteins, and cell cycle regulators. Sustained NF‐κB activation promotes tumorigenesis by fostering a pro‐inflammatory microenvironment, enhancing cellular proliferation, inhibiting apoptosis, and contributing to immune evasion.^44^, ^45^

Melatonin, which exhibits pleiotropic regulatory functions, has been shown to modulate both mitochondrial‐mediated apoptosis and NF‐κB‐dependent transcriptional activity. Mechanistically, melatonin inhibits phosphorylation of IκBα, thereby preventing NF‐κB nuclear translocation and suppressing transcription of target genes involved in inflammation and cell survival. Simultaneously, melatonin preserves mitochondrial integrity by reducing oxidative stress, stabilizing mitochondrial membranes, and attenuating cytochrome c release. These actions collectively result in the downregulation of caspase‐3 activation and suppression of pro‐survival signaling, thereby restoring the balance between cell survival and programmed cell death under oxidative or neoplastic stress.^46^

In rodent models, environmental stimuli capable of inducing oxidative stress—such as cigarette smoke and ionizing radiation—result in elevated ROS production, activation of pro‐inflammatory pathways, and direct DNA damage. Passive smoking exposure in rats led to increased expression of inflammatory cytokines (IL‐6, IL‐1β, and TNF‐α), reduced expression of anti‐apoptotic proteins (Bcl‐2 and Bcl‐xL), and increased levels of apoptotic markers (Bax and caspase‐3), along with upregulation of oncogenic genes such as VEGF, CYP1A1, and CYP1B1. Notably, melatonin administration mitigated these alterations, underscoring its protective effect on genetic material and its modulatory role in tumor‐related signaling pathways.^41^

Similarly, melatonin supplementation in rats subjected to total body irradiation significantly reduced hepatic DNA damage, as assessed by the comet assay. Melatonin also inhibited NF‐κB p65 and caspase‐3 expression while activating mitochondrial antioxidant pathways mediated by SIRT1 and PGC‐1α, both of which are crucial regulators of mitochondrial biogenesis and cellular metabolism. These findings reinforce the hypothesis that melatonin plays a pivotal role in maintaining genomic stability under conditions of severe oxidative stress, primarily through its antioxidant and antiproliferative properties.^47^

Melatonin also directly modulates genes involved in the cell cycle, DNA repair, and apoptosis. In a study investigating cisplatin‐induced ovarian toxicity, co‐administration of melatonin reduced lipid peroxidation, DNA fragmentation, and follicular apoptosis in mice. The expression of γ‐H2AX—a sensitive marker of DNA double‐strand breaks—was significantly lower in melatonin‐treated animals, indicating enhanced genomic integrity. Additionally, melatonin preserved ovarian reserve, underscoring its protective potential against genotoxic agents.^48^

In tumor‐bearing models, the genotoxicity induced by B16F10 melanoma cells and the protective role of melatonin in C57Bl/6 mice were evaluated. Tumor presence induced widespread DNA damage across multiple tissues (peripheral blood, liver, cerebral cortex, and spinal cord) and increased micronucleus frequency in bone marrow. Continuous melatonin administration for 60 days significantly reduced genotoxic damage and mutagenicity, attributable to its antioxidant, antiproliferative, and pro‐apoptotic effects. Although no protective effect was observed in the lungs (as measured by the comet assay), this may reflect undetected tumor apoptosis within that tissue.^49^

Furthermore, physical restraint stress partially abolished melatonin's antitumor effects in a DMBA‐induced breast cancer model. Mice subjected to both stress and carcinogen exposure exhibited increased ROS levels, reduced activity of antioxidant enzymes (SOD, GPx, and catalase), and heightened DNA damage, alongside increased expression of the proliferative marker PCNA. By contrast, melatonin alone effectively suppressed tumor growth, lipid peroxidation, and oxidative imbalance. These findings highlight the vulnerability of melatonergic pathways to environmental and behavioral stressors impacting the neuroendocrine axis.^50^

**FIGURE 5:**
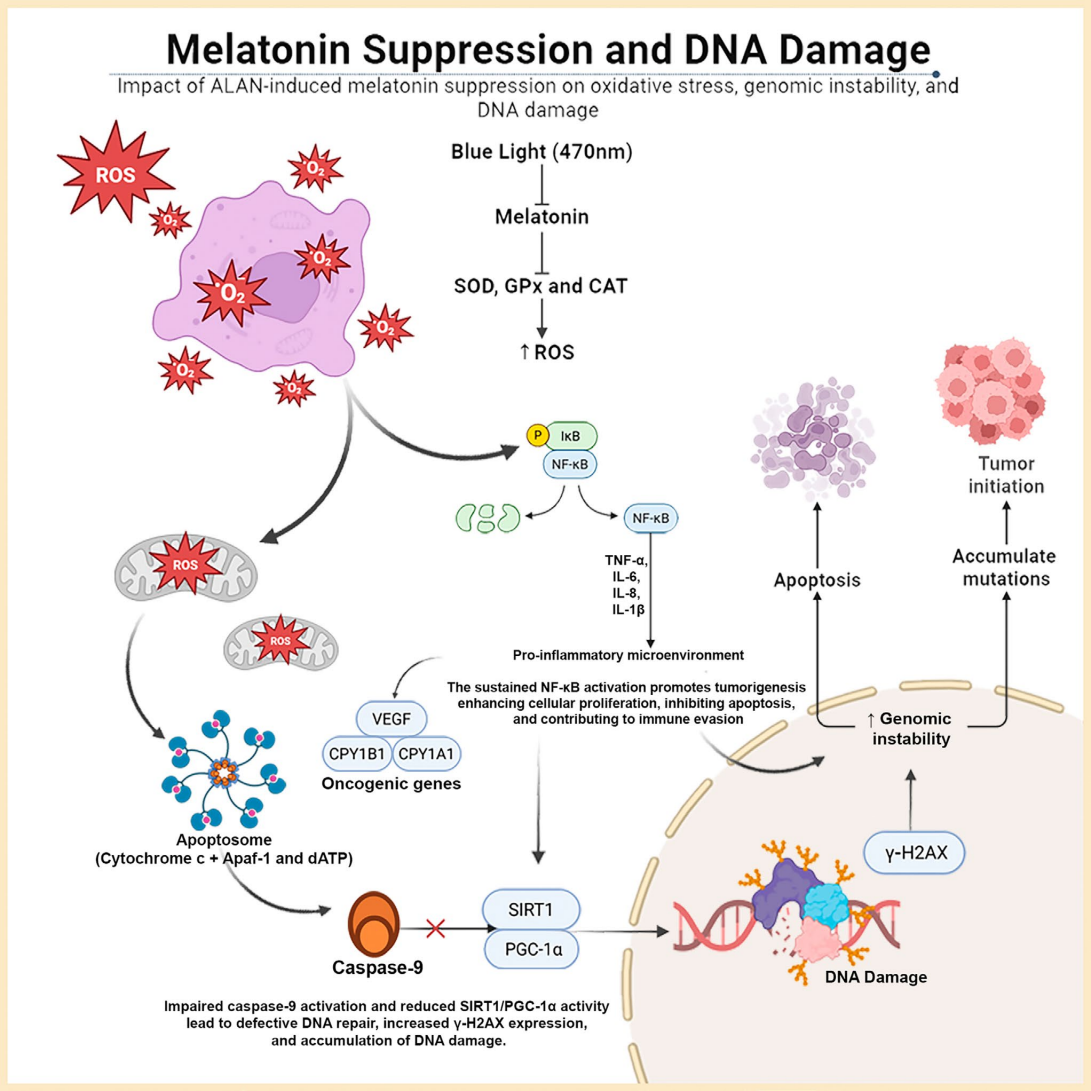
Melatonin suppression, oxidative stress, and DNA damage induced by exposure to artificial light at night (ALAN). This figure illustrates effects of blue light (470 nm) exposure on melatonin suppression and its downstream impact on oxidative stress, genomic instability, and DNA damage. The reduction in melatonin decreases the activity of antioxidant enzymes (SOD, GPx, and catalase), resulting in the accumulation of reactive oxygen species (ROS). Increased ROS production induces mitochondrial dysfunction, leading to cytochrome c release and apoptosome formation (Apaf‐1 and dATP). However, impaired caspase‐9 activation, along with reduced SIRT1/PGC‐1α activity, contributes to defective DNA repair and accumulation of γ‐H2AX, a marker of DNA damage. In parallel, NF‐κB signaling is activated, driving the expression of pro‐inflammatory cytokines (TNF‐α, IL‐6, IL‐8, IL‐1β) and oncogenic genes (VEGF, CYP1B1, CYP1A1). This creates a pro‐inflammatory tumor microenvironment, promoting genomic instability, inhibition of apoptosis, accumulation of mutations, and tumor initiation. Apaf‐1, apoptotic protease‐activating factor 1; CAT, catalase; CYP1A1, cytochrome P450 family 1 subfamily A member 1; CYP1B1, cytochrome P450 family 1 subfamily B member 1; dATP, deoxyadenosine triphosphate; GPx, glutathione peroxidase; IL‐1β, interleukin 1 beta; IL‐6, interleukin 6; IL‐8, interleukin 8; NF‐κB, nuclear factor kappa B; PGC‐1α, peroxisome proliferator‐activated receptor gamma coactivator 1‐alpha; ROS, reactive oxygen species; SIRT1, silent information regulator 1; SOD, superoxide dismutase; TNF‐α, tumor necrosis factor‐alpha; VEGF, vascular endothelial growth factor; γ‐H2AX, histone variant H2AX phosphorylated at serine 139.

In light of these findings, melatonin is confirmed as a potent oncostatic agent, regulating redox homeostasis, protecting genetic material, and promoting selective apoptosis in tumor cells. Chronic suppression of melatonin—such as that induced by sustained ALAN exposure—compromises genomic integrity and facilitates malignant transformation through cumulative mutations, persistent inflammatory microenvironments, and impaired immune surveillance. These mechanisms are schematically illustrated in Figure 5.

### Study limitations

This study analyzed research on the relationship between melatonin's oncostatic potential, ALAN, and carcinogenesis. Most of the evidence focuses on hormone‐dependent tumors, such as breast and prostate cancer, although associations have also been reported for other types of cancer, including thyroid cancer and childhood leukemia. While the proposed mechanisms are widely supported by molecular and genetic evidence, some discrepancies and gaps still need to be addressed to solidify these associations.

Although the reviewed studies provide important evidence regarding the relationship between ALAN and carcinogenesis, some limitations must be highlighted. First, methodological heterogeneity is a concern, as the methods for assessing ALAN exposure vary widely, including differences in intensity, duration, and light spectrum analyzed. Additionally, many studies focus on specific populations, such as night‐shift workers, which may limit the generalizability of the results to other contexts.

Another important aspect is the influence of potential confounding factors, such as genetic predisposition, dietary habits, and age, which are often not rigorously controlled in studies. Finally, although molecular mechanisms are well described in experimental studies, translating these findings into clinical practice still requires validation in large‐scale population studies. Works such as those of El‐Zaemey et al. illustrate that not all associations suggested by experimental studies are reflected in clinical markers, such as mammographic density in the case of breast cancer.^9^

## CONCLUSION

This systematic review highlights the complex relationship between circadian dysregulation, exposure to artificial light at night (ALAN), shift work, and carcinogenesis, with an emphasis on breast and prostate cancers. The analyzed studies suggest that melatonin suppression, induced by factors such as ALAN and night‐shift work, is a central mechanism affecting essential biological processes, including cell cycle regulation, DNA repair, and immune, inflammatory, and oxidative surveillance, creating a favorable environment for tumor development.

The findings demonstrate the influence of circadian genes and epigenetic factors on carcinogenesis, with alterations in the methylation of genes such as *DLX5*, *IGF2AS*, and *TP73* being associated with an increased risk of neoplasia. ALAN, particularly in the blue‐light spectrum, has been strongly linked to hormonal and oxidative dysregulation, cellular proliferation, and an increased risk of metastasis. Additionally, population‐based studies suggest that high levels of ALAN exposure, particularly in urban areas, are associated with a higher incidence of breast cancer, especially in premenopausal women and smokers.

Beyond its role in circadian rhythm regulation, melatonin has demonstrated oncostatic properties, with therapeutic potential to mitigate the effects of ALAN and shift work on carcinogenesis. Interventions such as exogenous melatonin administration and reduced ALAN exposure may represent promising strategies for the prevention and treatment of tumors associated with circadian disruption.

## Data Availability

Data sharing not applicable to this article as no datasets were generated or analysed during the current study.
